# The case-crossover design via penalized regression

**DOI:** 10.1186/s12874-016-0197-0

**Published:** 2016-08-22

**Authors:** Sam Doerken, Maja Mockenhaupt, Luigi Naldi, Martin Schumacher, Peggy Sekula

**Affiliations:** 1 Institute for Medical Biometry and Statistics, Faculty of Medicine and Medical Center - University of Freiburg, Freiburg, Germany; 2 Dokumentationszentrum schwerer Hautreaktionen (dZh), Medical Center, University of Freiburg, Freiburg, Germany; 3 USC di Dermatologia, Azienda Ospedaliero Papa Giovanni XXIII, Bergamo, Italy

**Keywords:** Case-crossover design, Lasso, Conditional logistic regression, Penalized regression, Severe cutaneous adverse reactions

## Abstract

**Background:**

The case-crossover design is an attractive alternative to the classical case–control design which can be used to study the onset of acute events if the risk factors of interest vary in time. By comparing exposures within cases at different time periods, the case-crossover design does not rely on control subjects which can be difficult to acquire. However, using the standard method of maximum likelihood, resulting risk estimates can be heavily biased when the prevalence to risk factors is very low (or very high).

**Methods:**

To overcome the problem of low risk factor prevalences, penalized conditional logistic regression via the lasso (least absolute shrinkage and selection operator) has been proposed in the literature as well as related methods such as the Firth correction. We apply and compare several penalized regression approaches in the context of a case-crossover analysis of the European Study of Severe Cutaneous Adverse Reactions (EuroSCAR; 1997–2001).

**Results:**

Out of 30 drugs, standard methods only correctly classified 17 drugs (including some highly implausible risk estimates), while penalized methods correctly classified 22 drugs.

**Conclusion:**

Penalized methods generally yield better risk classifications and much more plausible risk estimates for the EuroSCAR study than standard methods. As these novel techniques can be easily implemented using available R packages, we encourage routine use of penalized conditional logistic regression for case-crossover data.

**Electronic supplementary material:**

The online version of this article (doi:10.1186/s12874-016-0197-0) contains supplementary material, which is available to authorized users.

## Background

The case–control design is a common study design for assessing risk factors in epidemiology. However, recruiting suitable controls is a constant challenge [1]. For such settings, case series designs have been developed. One such type of case series is the case-crossover design [2] where every subject or patient serves as his own control. The association between the event of interest (e.g. disease onset) and risk factors is estimated by comparing exposure during the time period just prior to the event of interest to the same subject’s exposure during a reference period. The selection bias that case–control studies suffer from due to having to select controls is thus avoided by the case-crossover design. It also removes confounding effects from any time-invariant factors (e.g. sex, ethnicity).

Still, case-crossover studies face many of the remaining challenges that also affect case–control studies. In case–control studies, risk factors with very low (or very high) prevalence are problematic, e.g. they may result in unstable or unreliable estimates. Analogously, in case-crossover studies, risk factors become problematic if they are not very time-variant, i.e. only few subjects switch between exposed and unexposed during the observation period, leading consequently to few discordances. Such data may be referred to as sparse. A second common challenge is a potentially large number of risk factors under investigation. Furthermore, collinearity of risk factors is a typical problem. Many studies suffer from a combination of these three problems. As a result, estimation using standard maximum likelihood methods may be heavily biased or may not even be possible at all.

Penalized regression methods (also known as shrinkage, regularization, or sparse regression) have become popular and been applied plentiful since they are particularly suitable to remedy the challenges of collinear or sparse data [3, 4]. They use a modified likelihood, allowing estimation in instances where maximizing the original likelihood is not numerically possible.

Conditional logistic regression (CLR) using maximum likelihood is the standard method for the analysis of case-crossover studies; Avalos et al. [5] suggested adapting penalized methods to the CLR model and applied their methods successfully to both simulated and real data; they provided further applications in Avalos et al. [6] In our article, we aim to further investigate the use of penalized regression in case-crossover studies. We will include the same methods that were evaluated in Avalos et al. [5]. We do this using data from EuroSCAR [7], a study on the very rare and severe cutaneous adverse reactions Stevens-Johnson syndrome and toxic epidermal necrolysis (SJS/TEN) that are often drug induced.

The EuroSCAR study is attractive for this purpose for three reasons: firstly, it may potentially benefit from a penalization approach since it suffers from some of the challenges mentioned earlier. Secondly, even though EuroSCAR was a case–control study, here we only use data from cases, but are then able to compare our results to the results of the original case–control analysis, using the latter as a benchmark. And thirdly, a successor study to EuroSCAR is now running a registry of SJS/TEN patients, thereby only includes cases, which makes an assessment of case-crossover methods in this setting is highly relevant. We argue that penalized regression, due to the benefits in estimation and ease of implementation, should be encouraged for routine use for the case-crossover design.

## Methods

### Study design

**Fig. 1:**
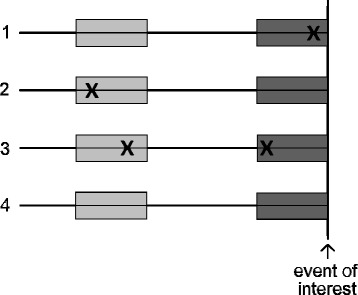
Illustration of the timeline of the case-crossover design, shown for four hypothetical subjects with respect to one risk factor. The *dark gray* area just prior to the event of interest marks the case period, the *light gray* area marks the reference period. Exposure to the risk factor is marked by “x”. Note that subjects 1 and 2 are discordant (differing exposures between case and reference periods), whereas subjects 3 and 4 are concordant (same exposures in case and reference periods)

The motivation behind the case-crossover design is as follows: if an exposure is prevalent right before the onset of an event of interest (e.g. disease onset) but absent during other times, it is natural to suspect that the exposure may be a trigger of the event. As opposed to a case–control design, the case-crossover design relies exclusively on cases in order to make inferences about risk factors. Within every case, the exposure to a time-varying risk factor during a time interval immediately prior to the event of interest, referred to as the case period, is compared to the exposure during a different, independent time window prior (or possibly also after) the event, referred to as the reference period (see Fig. 1 for an illustration). The case-crossover design is therefore most suitable for studying acute events. Since case and reference periods are compared within the same subject, potential time-invariant confounders have no influence. This is true for both known and unknown confounders. The design is attractive because it spares the difficulty of having to recruit suitable controls.

Case-crossover was evaluated in the context of SJS/TEN already in an earlier study [8]. The authors reported good efficiency of the case-crossover estimates for the risk factors under investigation, but with a few exceptions.

Since the case and reference periods within every subject are matched, data from a case-crossover study can be analyzed like a conventional matched case–control study using CLR.

### Statistical analysis

For our case-crossover risk estimation, we include the same methods that were suggested by Avalos et al. [5]. In addition, we considered two further methods not treated by them, namely sublasso and Firth correction.

#### Univariable conditional logistic regression

In a 1–1 matched study of *N* pairs (in the case-crossover setting, “pair” denotes a subject’s case period that is matched with his reference period), an estimate of a risk factor using univariable CLR is obtained through the log-likelihood function, which writes as

$$ \ell \left(\beta \right)={\displaystyle \sum_{n=1}^N}{\ell}_n\left(\beta \right)={\displaystyle \sum_{n=1}^N}\left[\beta {x}_{1n}- \log \left( \exp \left(\beta {x}_{1n}\right)+ \exp \left(\beta {x}_{0n}\right)\right)\right], $$

where x_1n_ denotes the exposure during the case period and x_0n_ the exposure during the reference period in the *n*th subject, and *β* is the regression coefficient [9]. The principle of maximum likelihood, which is most commonly used, states to use $\hat{\beta}$ as an estimate of *β* which maximizes the log-likelihood, i.e.

$$ \hat{\beta}=\underset{\beta }{\mathrm{argmax}}\ l\left(\beta \right). $$

The odds ratio (OR) of exposure to a risk factor can be directly obtained from a logistic regression model through $\exp \left(\hat{\beta}\right)$. For risk factor analysis in the case-crossover study of EuroSCAR, we fit separate univariable CLR models for every risk factor.

#### Multivariable conditional logistic regression

In contrast to the univariable model with just one risk factor and one estimate *β*, the multivariable model considers a vector ***β*** = (*β*_1_, …, *β*_*p*_)^*T*^ of *p* risk factors with exposure vectors ***x***_1***n***_ and ***x***_0***n***_ of length *p* in the log-likelihood function, using vector multiplication accordingly [9]. This allows for the simultaneous estimation of all risk factors in one model, thereby adjusting their effects for each other.

#### Lasso

**Fig. 2:**
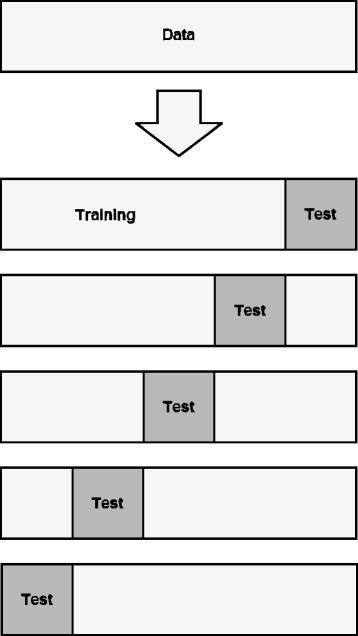
Illustration of cross-validation. The data is randomly split into K evenly-sized blocks (in this Figure, K = 5), partitioning the data into a training set and a test set. For each fold, a model is fit on the training set and evaluated on the test set. The model that performs best (i.e. has the highest likelihood) on average over all K folds is the one that is selected

A popular penalization method is the least absolute shrinkage and selection operator (lasso) [10]. Its popularity stems from its low computation cost and it has the property that as estimates are penalized, some are set to exactly 0. This is the main attraction of the lasso: it performs estimation and variable selection simultaneously. Estimation is based on the penalized log-likelihood

$$ \hat{\boldsymbol{\beta}}\left(\lambda \right)=\underset{\boldsymbol{\beta}}{\mathrm{argmax}}\ \left(l\left(\boldsymbol{\beta} \right) - \lambda {\displaystyle \sum_{j=1}^p}\left|{\beta}_j\right|\right) $$

where a penalty term is added to the log-likelihood that is tuned with a penalization parameter *λ ≥ 0*. The parameter λ controls the complexity of the model: if *λ = 0*, estimation is the same as with multivariable CLR, but as *λ* → ∞, estimates are shrunk and eventually set to 0. To determine an optimal value of *λ*, *K*-fold cross-validation is used. For this, the observations of the dataset are split into *K* evenly-sized blocks (while preserving the matching of the pairs). Given a value of *λ*, estimates are obtained by fitting a lasso model to the dataset with observations from one of the *K* blocks removed (the training set); the likelihood is then evaluated using observations from the left-out block (the test set, see Fig. 2 for clarification). This cross-validation step is performed *K* times, using every block once for evaluation. Typically, *K* = 5 or *K* = 10 is chosen; here, we use *K* = 10. The likelihood contributions from the K cross-validation steps are then summarized to obtain a likelihood given the value of *λ*. If this procedure is performed for a range of values for *λ*, we then choose the value for *λ* which maximizes the cross-validation likelihood.

#### Elastic net

A common alternative to the lasso is the elastic net [10]. The penalized log-likelihood

$$ \hat{\boldsymbol{\beta}}\left({\lambda}_1,{\lambda}_2\right)=\underset{\boldsymbol{\beta}}{\mathrm{argmax}}\ \left(l\left(\boldsymbol{\beta} \right) - {\lambda}_1{\displaystyle \sum_{j=1}^p}\left|{\beta}_j\right| - {\lambda}_2{\left({\displaystyle \sum_{j=1}^p}{\beta}_j^2\right)}^{\frac{1}{2}}\right) $$

contains two penalty terms that are tuned via parameters *λ*_*1*_*,λ*_*2*_ ≥ 0. If *λ*_*2*_ = 0, $\hat{\boldsymbol{\beta}}$ becomes the lasso estimate; if *λ*_*1*_ = 0, $\hat{\boldsymbol{\beta}}$ is the estimator of the ridge regression [10]. Elastic net is therefore a compromise between the lasso and ridge regression. Estimation of $\hat{\boldsymbol{\beta}}$ is again done through K-fold cross-validation, with the extension that maximization is done over a grid of values for *λ*_*1*_ and *λ*_*2*_, thereby increasing computation cost. The benefit of using the elastic net is that it tends to be better than lasso in selecting important variables when high correlations are present.

#### Bolasso

In general, the lasso selects all the relevant variables but additionally also a few irrelevant ones. As a remedy, Bach suggested the bolasso [11]. With the bolasso, a number of bootstrap samples are drawn from the dataset, where each bootstrap sample is generated by sampling N pairs from the dataset with replacement. Here, we will draw 1000 bootstrap samples. A lasso model is then fit to each bootstrap sample. Risk factors not selected in at least 75 % of the bootstrap samples are set to zero, the remaining risk factor estimates are set to their average estimates from all the bootstrap samples. Using a threshold of 75 % had the best results in Avalos et al. [5] and was thus used by us.

#### Sublasso

Analogous to the bolasso method, but instead of drawing bootstrap samples, sublasso uses subsampling by drawing 75 % of the observations without replacements. Though sublasso was not part of the investigation by Avalos et al. [5], we included it here as an ad hoc extension because there are many contentions to favor subsampling over bootstrapping [12] and because bolasso is easily extendable to accommodate sublasso.

#### Random lasso

Similar in name and principle to random forest, random lasso is an extension of bolasso which consists of two steps [13]. The first step is the same as the bolasso procedure, but instead of including all risk factors, for each bootstrap sample only a random subset of size *q*_*1*_ of the risk factors are used for fitting. The resulting estimates then serve as weights in step two: lasso is again fitted to bootstrap samples by only considering a random subset of size *q*_*2*_ of the risk factors, where the risk factors are selected proportionally to the weights determined in the first step. In the EuroSCAR study, *p = 30*, therefore we select the optimal *q*_*1*_ and *q*_*2*_ from the set 15, 20, 25 and 30 using cross-validation. As in bolasso, variables are set to their average estimate from all bootstrap samples if their selection is at least 75 %.

#### Firth correction

Similar to the lasso, Firth [14] suggested maximizing a modified log-likelihood function,

$$ \hat{\boldsymbol{\beta}}=\underset{\boldsymbol{\beta}}{\mathrm{argmax}}\ \left(l\left(\boldsymbol{\beta} \right) + \frac{1}{2} \log \left( \det I\left(\boldsymbol{\beta} \right)\right)\right), $$

where *I*(***β***) is the observed information matrix, to overcome the challenge of data separation when sample size is small. Firth correction is another effective bias-correction method which has gained some popularity. It was not used by Avalos et al. [5] but it has shown good results in a study design very similar to case-crossover [15]. The adaptation of the Firth correction for CLR is described by Heinze & Puhr [16] and Sun et al. [17].

Analyses were performed using R version 3.1.2. To calculate correlations of risk factors, we employ the Pearson/Spearman correlation, the two being equivalent for binary data such as encountered here. Standard CLR was done using the R package survival. CLR with lasso penalization was performed using the R package clogitL1 by Reid & Tibshirani [18] which is described in Reid & Tibshirani [19] and is available at CRAN. Alternative R packages for CLR with lasso are clogitLasso, also available at CRAN, which is described in Avalos & Pouyes [20], and in more detail in Avalos et al. [21], and pclogit [22] available at www.columbia.edu/~sw2206/softwares.htm. To implement the Firth correction, we used a macro CFL by Heinze available at http://cemsiis.meduniwien.ac.at/en/kb/science-research/software/statistical-software/fllogistf/ which was run using SAS version 9.2. The package coxphf [23] provides an implementation of the Firth regression in R for the Cox model. Since the likelihood for a CLR model is equivalent to that of a Cox model with a particular data structure, this package is a suitable alternative. Our R and adapted SAS scripts are available in the supplementary material.

### Study population

We apply the methods under investigation using patient data from the EuroSCAR study [7]. The study was designed as a multinational case–control study on patients with SJS/TEN. As these events are mainly caused by a variety of drugs, the main aim was to assess the risk of drugs or drug groups.

The recruitment of cases and controls took place in six countries (Austria, France, Germany, Israel, Italy, and The Netherlands) within the period from April 1997 to December 2001. Altogether, 379 cases and 1,505 controls were included. From the obtained data, 30 drug groups (henceforth simply referred to as drugs) were defined as either “highly suspected” (*n* = 9), “suspected” (*n* = 10), or not suspected (“other”, *n* = 11) of causing SJS/TEN. These classifications were corroborated by Papay et al. [24]. For more details on the EuroSCAR study, we refer the reader to Mockenhaupt et al. [7].

For the case-crossover analysis, only the 379 cases of the EuroSCAR study were extracted. 22 patients were excluded because they had not taken any of the 30 relevant drugs during the observation period and thus could not contribute any information to the estimations. A further 6 patients were excluded because their observation period was too short to accommodate the minimum required number of days for both the case and reference period, leaving a total of 351 subjects for our analysis. The data used in our analysis is available in the supplementary material (see Additional file 1).

### Evaluation of methods

In our work, the goal of the competing methods is to perform a new classification, herein called reclassification, of the drugs that would ideally resemble the classifications of the case–control study of EuroSCAR [7], thus the latter will serve as a benchmark. Using solely the estimates of the case–control study (Additional file 2: Table S1), the best resemblance of risk classes is obtained by deeming drugs “highly suspected” if log OR > 2.4, “suspected” if 0.45 < log OR < 2.4 and “other” if log OR < 0.45. Further, we employ receiver operating characteristic (ROC) curves by discriminating which drugs are correctly reclassified as “highly suspected”, and alternatively by discriminating which drugs are correctly reclassified as “highly suspected” or “suspected”.

## Results

Correlations of drugs within case periods, and separately within reference periods, were all considerably low (<0.32 in absolute value).

Estimates from a multivariable CLR, arguably the most common technique for analyzing matched data, suffer from drugs that are prevalent in subjects’ case periods but infrequent in the reference periods, or vice versa. Only cases with discordant exposure in both periods contribute to the likelihood, thus drugs with only few discordances prove to be problematic for analysis. For such drugs, resulting risk estimates tend to be implausible, either because they are too large or too small.

**Fig. 3:**
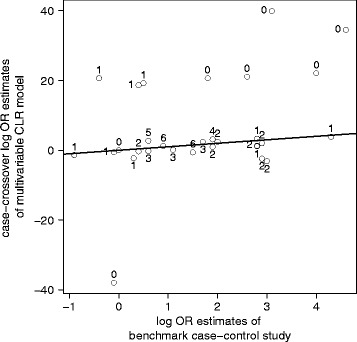
Estimates from the multivariable CLR plotted against the estimates from the case–control study (Mockenhaupt et al. [7]). The line marks the 45-degree line of equality (note the different scales on the x- and the y-axis). Ideally, the estimates from the multivariable CLR would resemble the estimates from the case–control study; however, some very large discrepancies are apparent. Displayed next to the estimates is the minimum of the two possible discordances between case and reference period of the risk factor. It can be seen that drugs with extreme estimates have only one or zero such discordances, while drugs with at least two discordances have plausible estimates

Figure 3 displays the estimates of the multivariable CLR model plotted against the estimates from the benchmark case–control study. Ideally, the estimates of the former would agree with the latter, in which case the plotted points would fall on the 45 degree line. However, there are some very large disagreements between the two. As indicated by the numbers next to the plotted points, it appears to occur only (though not necessarily) for drugs with infrequent discordances. Therefore, multivariable CLR is very unsatisfactory for obtaining plausible risk estimates.

**Fig. 4:**
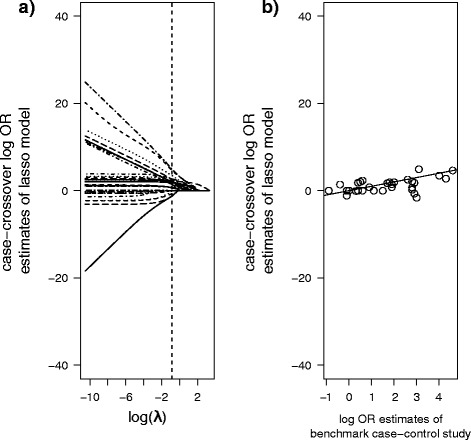
**a** Estimates of the lasso for different values of λ. As λ increases, risk estimates shrink and are eventually 0. The dashed line marks the optimal λ-value and the corresponding optimal estimates, as determined by cross-validation. **b** Similarly to Fig. 3, the optimal lasso estimates are plotted against the benchmark case–control estimates. Agreement between the two is evidently much better than for multivariable CLR. The line marks the 45-degree line of equality. The y-scale is chosen to accommodate comparisons with Fig. 3

Figure 4a shows the risk estimate paths of the lasso for different values of the tuning parameter *λ*. As the tuning parameter increases, the risk estimates shrink and are eventually zero. Cross-validation determines the optimal value of λ and is marked by the vertical dashed line (see Additional file 2: Figure S1 for illustration). These optimal risk estimates are shown in Fig. 4b. They agree much better with the estimates of the benchmark case–control study. There no longer is a problem of highly implausible values.

Table 1 is a summary of the number of correctly reclassified drugs according to the classification by Mockenhaupt et al. [7] (see Additional file 2: Table S2 for a detailed version). Multivariable CLR performs worst overall among all the methods and only correctly reclassifies 17 out of 30 drugs. The penalized methods show large improvements, with lasso and random lasso performing best, and similar results from elastic net, sublasso and Firth correction.

**Table 1:** Number of drugs correctly identified under new reclassification rule by each method

Original Classification Rule for new reclassification	Highly suspected (*n* = 9) log OR > 2.4	Suspected (*n* = 10) 0.45 < log OR < 2.4	Other (*n* = 11) log OR < 0.45	Total (*n* = 30)
Univariable CLR	5	7	7	19
Multivariable CLR	6	2	9	17
Lasso	5	8	9	22
Elastic net	4	8	9	21
Bolasso	4	6	9	19
Sublasso	4	8	9	21
Random lasso	6	6	10	22
Firth correction	5	8	7	20
Case-control^a^	9	10	8	27

^a^Note that even though the case–control study was the basis for the risk classification in Mockenhaupt et al. [7], its estimates do not fully agree with the reclassification scheme used by us. This is because experts classified some of the drugs differently than its risk estimates would suggest based on their experience and opinion, whereas we employ a reclassification scheme using only the estimates

The arguably most important aspect of the EuroSCAR study was to correctly identify drugs that are highly suspected of causing SJS/TEN. This poses a binary discrimination problem (“highly suspected” versus “suspected” or “other”) for which we use ROC curves that do not rely on a single fixed cut-off value to discriminate between risk classes.

**Fig. 5:**
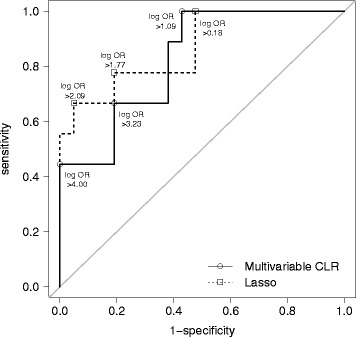
ROC curves of multivariable CLR and the lasso for reclassifying highly suspected drugs. Cut-off values of the log ORs for discriminating between highly suspected drugs versus suspected or other drugs range from 0 to 4; the sensitivity and 1-specificity of several cut-off values are highlighted. A 45-degree line of equality is added for reference

Figure 5 shows a comparison of ROC curves for multivariable CLR and the lasso. Note that due to the shrinkage of its estimates, the lasso uses a very different discrimination scheme: where multivariable CLR and the lasso have the same or similar specificity, the lasso uses a much lower log OR cut-off. It can be seen that the ROC curve of lasso is largely superior to the ROC curve of the multivariable CLR model. Therefore, the lasso outperforms multivariable CLR in discriminating highly suspected drugs.

Summarizing ROC curves for all methods with their area under the curve, Table 2a shows that lasso, elastic net and Firth correction perform best for correctly identifying highly suspected drugs. Multivariable CLR again performs worst, and the resampling methods fall in-between.

**Table 2:** Area under the curve for binary discrimination of drugs

	a) AUC for “highly suspected drugs“	b) AUC for “highly suspected drugs“ or “suspected drugs“
Univariable CLR	0.836	0.871
Multivariable CLR	0.741	0.775
Lasso	0.862	0.861
Elastic net	0.868	0.909
Bolasso	0.804	0.813
Sublasso	0.815	0.823
Random lasso	0.820	0.847
Firth correction	0.852	0.928
Case-control^a^	0.947	0.809

^a^see footnote in Table 1

We also look at which methods best discriminate between risk classes “highly suspected” or “suspected” versus “other”. Again the lasso performs better overall than multivariable CLR. Among all methods, Table 2b shows that Firth correction and elastic net have the best ability to discriminate in this setting. The robustness of these results is corroborated by a sensitivity analysis wherein the AUCs were calculated for 100 subsamples drawn without replacement (Additional file 2: Figure S2). Interestingly, the benchmark case–control study performs rather poorly here. A possible explanation could be that in the case–control study by Mockenhaupt et al. [7] some drugs were classified as “other” simply because they were newly introduced, when in fact their estimates would suggest that they are “suspected” or “highly suspected”. Therefore, an assessment of the supposedly “correct” case–control risk estimates alone need not necessarily lead to the best discrimination scheme.

## Discussion

### Summary of our work

Penalized regression has been introduced in the literature as a method for bias-correction and has many applications; here, we argue in favor of it in the context of the case-crossover design. Similarly to a previous study, we evaluated the case-crossover design in the context of Stevens-Johnson syndrome and toxic epidermal necrolysis (SJS/TEN) [8]. Using data from the EuroSCAR study [7], the superiority of the lasso over multivariable conditional logistic regression (CLR) was apparent, since the latter seemingly suffered from the problem of sparse data.

We also investigated several resampling approaches together with penalization methods. They did not demonstrate any improvement, thus our results suggest that the increased computation cost is not justified. The Firth correction performed reasonably compared to the penalized methods and was even best in discriminating “highly suspected” or “suspected” drugs. This is particularly noteworthy when considering that the other penalized methods tune their penalty terms whereas the Firth correction uses a constant factor of 0.5. It may be an interesting investigation to explore if a similarly tuned Firth correction would be an appropriate extension.

There are several limitations to our study. First and foremost, although we enjoyed the advantage of having benchmark case–control estimates for our case-crossover analysis, the benchmark is not perfect since the original case–control study had its own limitations. Because of its limitations, some of the risk factors of the case–control study could be estimated only through univariable CLR. However, univariable estimates may not be the best benchmark for comparing multivariable methods. Also, when single cut-off values are used to compare all methods (as was done for Table 1), direct comparisons of estimators may not be suitable when some methods shrink estimates and some do not. For this reason, we also used ROC curves which do not rely on single cut-off values. Further, assessment solely based on estimates may be incomplete without also considering standard errors of the estimates. This, however, is a weakness of the lasso, as standard errors are not directly available [25, 26]. For a further discussion on the limitations of penalized regression, see Greenland et al. [27].

### Penalized regression in epidemiology and case-crossover

A good introduction to penalized regression is given by Cole et al. [4]. However, epidemiologic literature on penalized regression is still relatively scarce. An example of an exception is Rose [28] who developed risk scores for mortality prediction using, among other techniques, penalized regression. In another study, Burgette et al. [29] implement the lasso and elastic net to model adverse birth outcomes. Further, Smith et al. [30] apply penalized regression to model the BMI in a longitudinal study. In a large study that uses case-crossover with CLR, Mostofsky et al. [31] investigate the association between particle constituents of air pollution and health outcomes. Theirs is a great example where penalized regression could be applied since their risk factors suffer from high collinearity.

Sullivan & Greenland [32] point out that sparse-data artefacts often go unrecognized in study reports. They provide an example of a case–control study which reported an odds ratio between ever having smoked and ICU admission of 65 (95 % CI 6.3–672) without ever considering the plausibility of the estimate. Cole et al. [4] stress the importance of penalized regression by pointing out that

“[…] epidemiologists are aware of problems due to sparse-data bias in very small studies, but sparse-data bias appears to be less widely recognized when it occurs in larger studies.” (p. 257)

Among the attractive features in favor of penalized methods is that the shrinkage of coefficients is proportional to the estimated variance of the coefficients, thus unstable estimates are shrunk more than stable ones. And further, penalized regression can be applied when covariates are collinear and conventional methods fail completely [3].

In the context of case-crossover, there has been hardly any work on using penalized regression. Walter & Tiemeier [33] conducted a survey of 300 articles published in four major epidemiologic journals, not one of which used penalized regression. Though their work is no longer up to date, a recent literature search revealed only three articles that use penalized regression in the context of case-crossover, all by Avalos and her colleagues (Additional file 2: Table S3).

In a study design closely related to case-crossover, the self-controlled case series design, work in the literature on penalization methods has been equally scarce. The self-controlled case series is tantamount to the classical cohort study in the same way that case-crossover is the case series equivalent of a classical case–control study [34]. In a work using the self-controlled case series design, Zeng et al. [15] study bias correction methods for datasets with a small number of adverse events (i.e. a sparse data), such as a vaccine safety study. In it, the Firth estimate consistently outperformed the classical maximum likelihood estimate.

In a recent work by Avalos et al. [35], the authors describe in detail an algorithm more efficient than the one in their initial paper [5], making it suitable even for large datasets. The authors also provide a good comparison of the different R packages available that implement the lasso in CLR (and also Cox and unconditional logistic regression).

## Conclusion

Standard maximum likelihood is the default in most statistical software packages and has many desirable large-sample properties, among them asymptotic unbiasedness. The large-sample condition, however, is difficult to achieve satisfactorily, and consequently, bias can be substantial for small-sample studies.

Therefore, arguments are strong for wider use of penalized regression in epidemiological studies. Of great practical importance is that penalized regression is easily implemented for linear, Poisson, Cox, or CLR models. For comparison, running a CLR requires two lines of code in R:
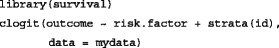


Running the lasso is only marginally more work, requiring three lines:
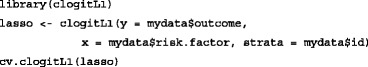


Avalos et al. [36] recommend penalized regression as an alternative to conventional strategies, and Cole et al. [4] even suggest that penalized likelihood should arguably replace standard maximum likelihood as the default method. For the case-crossover design, we also encourage penalized regression for routine use.

## Abbreviations

AUC, area under the curve; CLR, conditional logistic regression; EuroSCAR, European Study of Severe Cutaneous Adverse Reactions; Lasso, least absolute shrinkage and selection operator; OR, odds ratio; ROC, receiver operating characteristic; SJS/TEN, Stevens-Johnson syndrome and toxic epidermal necrolysis

## Additional files

Additional file 1: Dataset and scripts used for analysis. (ZIP 28.0 kb) Additional file 2: Table S1. Results for the 30 drugs and drug groups of the EuroSCAR study, a Multinational Case–control study in Europe and Israel between 1997 and 2001 on patients with SJS/TEN [4]. **Table S2.** Reclassification of individual drugs by each method. **Table S3.** Relevant results of literature search for penalized regression used with case-crossover studies, performed on November 21^st^, 2015. **Figure S1.** Deviance (−2 × log-likelihood) of the lasso for different values of λ using 10-fold cross-validation. **Figure S2.** Sensitivity analysis of Table 2a. (DOCX 121 kb)
