# Supplementary material for: The case-crossover design via penalized regression
Source: BMC Med Res Methodol. 2016 Aug 22;16(1):103. doi: 10.1186/s12874-016-0197-0 (PMC4994302; doi:10.1186/s12874-016-0197-0)
Supplement: Additional file 2: Table S1. — Results for the 30 drugs and drug groups of the EuroSCAR study, a Multinational Case–control study in Europe and Israel between 1997 and 2001 on patients with SJS/TEN [4]. Table S2. Reclassification of individual drugs by each method. Table S3. Relevant results of literature search for penalized regression used with case-crossover studies, performed on November 21st, 2015. Figure S1. Deviance (−2 × log-likelihood) of the lasso for different values of λ using 10-fold cross-validation. Figure S2. Sensitivity analysis of Table 2a. (DOCX 121 kb) [file 12874_2016_197_MOESM2_ESM.docx]

The case-crossover design via penalized regression (Additional file)

# Additional file 1

Table S1 Results for the 30 drugs and drug groups of the EuroSCAR study, a Multinational Case-control study in Europe and Israel between 1997 and 2001 on patients with SJS/TEN [4]. Note that the groups of “highly suspected drugs”, “suspected drugs” and “other” refer to the original classification of drugs as published in Mockenhaupt et al. [4].

|  | Log OR | OR | 95% CI |
| --- | --- | --- | --- |
| Highly suspected drugs |  |  |  |
| Allopurinol | 2.9 | 18 | 11 – 32 |
| Anti-infective sulfonamides: |  |  |  |
| -Cotrimoxazole | 4.6 | 102^1^ | 17 – 754 |
| -Other | 4.0 | 53^1^ | 7.0 – 410 |
| Carbamazepine | 4.3 | 72 | 23 – 225 |
| Lamotrigine | 2.6 | >14^2^ |  |
| Nevirapine | 3.1 | >22^2^ |  |
| Oxicam NSAIDs | 2.8 | 16 | 4.9 – 52 |
| Phenobarbital | 2.8 | 16 | 5.0 – 50 |
| Phenytoin | 2.8 | 17 | 4.1 – 68 |
| Suspected Drugs |  |  |  |
| Acetaminophen | 0.6 | 1.9 | 1.2 – 2.8 |
| Acetic acid NSAIDs | 1.7 | 5.6 | 2.6 – 12 |
| Acetylsalicyclic acid | 0.5 | 1.6 | 0.9 – 2.7 |
| Aminopenicillins | 0.9 | 2.4 | 1.0 – 5.9 |
| Cephalosporins | 2.0 | 7.3 | 2.4 – 22 |
| Corticosteroids | 1.5 | 4.5 | 2.4 – 8.7 |
| Macrolides | 1.9 | 6.8 | 2.6 – 18 |
| Pyrazolones | 1.1 | 3.1 | 1.2 – 7.7 |
| Quinolones | 1.9 | 6.9 | 1.8 – 27 |
| Tetracyclines | 1.8 | 6.3 | 1.6 – 25 |
| Other drugs |  |  |  |
| ACE inhibitors | -0.1 | 0.9 | 0.5 – 1.5 |
| Beta blocking agents | -0.1 | 0.9 | 0.5 – 1.5 |
| Calcium channel blockers | 0.3 | 1.4 | 0.8 – 2.4 |
| Furosemides | 0.6 | 1.8 | 0.9 – 3.4 |
| HMG-COA reductase inhibitors | -0.9 | 0.4 | 0.2 – 1.1 |
| Insulins | 0.0 | 1.0 | 0.3 – 3.3 |
| Propionic acid NSAIDs | 0.4 | 1.5 | 0.6 – 3.4 |
| Proton pump inhibitors: |  |  |  |
| -Pantoprazole | 2.9 | 18^1^ | 3.9 – 85 |
| -Other | 0.4 | 1.5 | 0.7 – 3.2 |
| Thiazide diuretics | -0.4 | 0.7 | 0.4 – 1.4 |
| Tramadol | 3.0 | 20^1^ | 4.4 – 93 |

^1^unadjusted estimate due to low exposure prevalence in controls (<3)

^2^exact lower 95% CI due to absence of any exposed controls

Table S2: Reclassification of individual drugs by each method. H: highly suspected (log OR > 2.4) S: suspected (0.45 < log OR < 2.4) O: not suspected (other, log OR < 0.45). Note that the groups of “highly suspected drugs”, “suspected drugs” and “other” refer to the original classification of drugs as published in Mockenhaupt et al. [4].

|  | Univariable CLR | Multivariable CLR | Lasso | Elastic  net | Bolasso | Sublasso | Random lasso | Firth correction | Case-control |
| --- | --- | --- | --- | --- | --- | --- | --- | --- | --- |
| Highly suspected drugs |  |  |  |  |  |  |  |  |  |
| Allopurinol | S | S | S | S | S | S | H | S | H |
| Anti-infective sulfonamides: |  |  |  |  |  |  |  |  |  |
| -Cotrimoxazole | H | H | H | H | H | H | H | H | H |
| -other | H | H | H | H | H | H | H | H | H |
| Carbamazepine | H | H | H | H | H | H | H | H | H |
| Lamotrigine | H | H | H | S | S | S | H | H | H |
| Nevirapine | H | H | H | H | H | H | H | H | H |
| Oxicam NSAIDs | S | S | O | O | O | O | O | S | H |
| Phenobarbital | S | S | S | S | S | S | S | S | H |
| Phenytoin | S | H | S | S | S | S | S | S | H |
| Suspected drugs |  |  |  |  |  |  |  |  |  |
| Acetaminophen | H | H | S | S | H | S | H | S | S |
| Acetic acid NSAIDs | S | H | S | S | S | S | S | S | S |
| Acetylsalicyclic acid | H | H | S | S | S | S | S | S | S |
| Aminopenicillins | S | S | S | S | S | S | S | S | S |
| Cephalosporins | S | H | S | S | H | S | H | S | S |
| Corticosteroids | S | O | O | O | O | O | O | S | S |
| Macrolides | S | H | S | S | S | S | S | S | S |
| Pyrazolones | S | O | O | O | O | O | O | S | S |
| Quinolones | S | S | S | S | S | S | S | S | S |
| Tetracyclines | H | H | S | S | S | S | S | S | S |
| Other drugs |  |  |  |  |  |  |  |  |  |
| ACE inhibitors | O | O | O | O | O | O | O | O | O |
| Beta blocking agents | O | O | O | O | O | O | O | O | O |
| Calcium channel blockers | O | O | O | O | O | O | O | O | O |
| Furosemides | S | O | O | O | O | O | O | S | S |
| HMG-COA reductase inhibitors | O | O | O | O | O | O | O | O | O |
| Insulins | O | O | O | O | O | O | O | O | O |
| Propionic acid NSAIDs | S | H | S | S | S | S | S | S | O |
| Proton pump inhibitors: |  |  |  |  |  |  |  |  |  |
| -Panzoprazole | O | O | O | O | O | O | O | O | H |
| -other | S | O | O | O | O | O | O | S | O |
| Thiazide diuretics | S | H | S | S | S | S | O | S | O |
| Tramadol | O | O | O | O | O | O | O | O | H |

Table S3: Relevant results of literature search for penalized regression used with case-crossover studies, performed on November 21^st^, 2015.

| Relevant results | Found by PubMed^1^ | Found by Web of Science^2^ |
| --- | --- | --- |
| [1] | yes | yes |
| [2] | yes | yes |
| [3] | no | yes |

^1^ www.ncbi.nlm.nih.gov/pubmed

Search query: (case-crossover) AND ((penali*) OR (shrink*) OR sparse OR lasso OR regulari*)

Results: 9

^2^ www.webofknowledge.com

Search query: TOPIC: (case-crossover) AND (TOPIC: (penali*) OR TOPIC: (shrink*) OR TOPIC: (sparse) OR TOPIC: (lasso) OR TOPIC: (regulari*))

Results: 20

[1] was the motivation for our current work whose investigated methods we adapted in order to compare results. [2] is a more applied treatment of the methods presented in [1]. [3] is a description of the R package clogitlasso.

# Figures

Figure S1: Deviance (-2×log-likelihood) of the lasso for different values of λ using 10-fold cross-validation. The minimum deviance is achieved for

log(λ) = -0.83 (vertical dashed line).


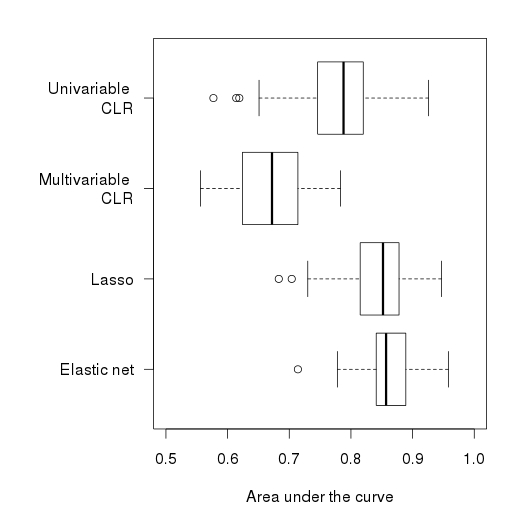


Figure S2: Sensitivity analysis of Table 2a. For 100 subsamples wherein 75% of the observations are drawn without replacement, the AUCs are calculated and illustrated with boxplots. The Firth correction is omitted due to large computational time.

# References

1. Avalos M, Grandvalet Y, Adroher ND, et al. Analysis of multiple exposures in the case-crossover design via sparse conditional likelihood. *Stat Med* 2012;31(21):2290-2302.
2. Avalos M, Orriols L, Pouyes H, et al. Variable selection on large case-crossover data: application to a registry-based study of prescription drugs and road traffic crashes. *Pharmacoepidemiol Drug Saf* 2014;23:140-151.
3. Avalos M, Grandvalet Y, Pouyes H, et al. High-dimensional sparse matched case-control and case-crossover data: A review of recent works, description of an R tool and an illustration of the use in epidemiological studies. *Computational Intelligence Methods for Bioinformatics and Biostatistics. Volume 8452. Edited by Formenti, E., Tagliaferri, R., Wit, E. Lecture Notes in Computer Science* 2014;109-124.
4. Mockenhaupt M, Viboud C, Dunant A, et al. Stevens-Johnson syndrome and toxic epidermal necrolysis: assessment of medication risks with emphasis on recently marketed drugs. The EuroSCAR study. *J Invest Dermatol* 2008;128(1):35–44.
