# Supplementary material for: The case-crossover design via penalized regression
Source: BMC Med Res Methodol. 2016 Aug 22;16(1):103. doi: 10.1186/s12874-016-0197-0 (PMC4994302; doi:10.1186/s12874-016-0197-0)
Supplement: Additional file 1: — Dataset and scripts used for analysis. (ZIP 28.0 kb) [file 12874_2016_197_MOESM1_ESM.zip › data/dataset_explained.doc]

```
COMMON INFORMATION
id		Num		patient number


EXPOSURE INFORMATION REGARDING DRUGS
(0 = not exposed, 1 = exposed)

A HIGHLY SUSPECTED DRUGS (9)
allo		Num		allopurinol
carb		Num		carbamazepine
cotr		Num		cotrimoxazole
lamo		Num		lamotrigine
nevi		Num		nevirapine
osul		Num		other sulfonamides
oxic		Num		oxicam nsaids
pheb		Num		phenobarbital
phet		Num		phenytoin

B SUSPECTED DRUGS (10)
acac		Num		acetic acid nsaids
acty		Num		acetylsalicylic acid
ampi		Num		aminopenicillin
ceph		Num		cephalosporins
macr		Num		macrolides
para		Num		acetaminophen/paracetamol
pyra		Num		pyrazolones
quin		Num		quinolones
ster		Num		corticosteroids
tetr		Num		tetracyclines

C OTHER DRUGS (11)
acei		Num		ACE inhibitors
beta		Num		beta blockers
ccbl		Num		calcium channel blockers
furo		Num		furosemides
hmg 		Num		HMG-CoA reductase inhibitors
insu		Num		insulins
oppi		Num		other proton pump inhibitors
pant		Num		pantoprazole
prop		Num		propionic acid nsaids
thia		Num		thiazide diuretics
tram		Num		tramadol

INFORMATION REGARDING PERIOD
(0 = reference period, 1 = case period)

outcome	Num		period
```
